# Progress in Medicine

**Published:** 1900-08-11

**Authors:** 


### Progress in Medicine.

### NEUROLOGY.

(*Continued from page* 303.)

**Insomnia.**—Broadbent[2] says that as a rule sedatives lead to the establishment of an evil habit, and to deterioration of the bodily and mental faculties. It is essential to discover the cause of the sleeplessness, and treat that. In anæmic patients cold feet at night may prevent sleep; for these a hot bottle and envelopment of the feet and legs in flannel should be used. In cases of sluggish circulation a little hot and strong beef-tea or hot milk should be taken on going to bed. In gout, rheumatoid arthritis, the feet may feel hot and burning; in such cases phenacetin or Dover's powder is useful. In sleeplessness due to high arterial tension the diet should be regulated, nitrogenous intake limited, and some form of mercurial pill given twice a week. In conditions of low arterial tension the patient is very drowsy when sitting or standing up, but wide awake when lying down. A tonic treatment is indicated. The most common cause of sleeplessness is indigestion, especially with gaseous distension of the stomach. It is highly characteristic of this form of sleeplessness that the patient is roused from the sleep into which he has fallen in the early hours of the morning and then remains awake. The treatment is that of dyspepsia with, in addition, a glass of hot water before undressing, or an alkaline carminative draught. Influenza may cause most obstinate sleeplessness, and may be complicated with dilatation of the stomach. If tonics, such as arsenic, phosphorus, strychnine, and quinine do not give speedy relief it is best to employ combinations of opium and hyoscyamus with carminatives. In the sleeplessness of alcoholic excess it is best to prescribe considerable doses of strychnine and, perhaps, digitalis. Bradbury[3] says that the bromides are useful in insomnia due to worry or overwork, when relief cannot be obtained by non-medical means. They are useless in the presence of pain. Chloral hydrate is more powerful, but is a depressant to the heart and vascular system, and should therefore be avoided in cardiac failure, and generally in diseases with low blood pressure, such as typhoid fever. In cases of high arterial pressure it may be given with impunity. Batyl chloral hydrate will relieve the insomnia due to neuralgia of the fifth nerve. Chloralamide is less depressing to the circulation and respiration than chloral hydrate. It may be given in doses of 30—40 grains. Chloralose is more powerful than chloral hydrate or chloralamide. It is said to have no action on the heart in therapeutic doses, and the blood pressure is slightly raised. It is specially recommended as a hypnotic in cardiac and dyspeptic troubles, but contra-indicated in chronic Bright's disease. It may be given in doses of from 3—6 grains in 1½ to 2 ounces of water. Paraldehyde is safer, and especially serviceable in asthma and heart disease. Sulphonal and trional are valuable hypnotics; they have no action on the circulatory or respiratory systems; they are slightly cumulative.

Opium and morphia are most useful when there is great pain. Their action on the heart and vascular system is small, so that in heart disease, especially if attended with pain, they may generally be employed; but their influence on the respiration is well marked, and in all respiratory diseases accompanied by impeded respiration from excessive secretion they should be regarded with disfavour. Hyoscin is one of the most powerful hypnotics, and is one of the best substitutes for morphia we possess. One two-hundredth to one one-hundred-and-fiftieth of a grain of the hydrobromide hypodermically is usually sufficient to induce sleep. Cannabis indica is given in migraine and neuralgia, and often alleviates the pain of gastralgia.

**Degeneration in the Nervous System.**—In his fourth Croonian lecture, Mott[4] discussed the pathology of general paralysis of the insane, which he defined as a progressive degeneration affecting especially the association neurones of the frontal and central convolutions of the cerebral hemispheres. General paralysis and locomotor ataxy are both a primary and progressive decay of nervous elements, affecting different parts of the nervous system. It is the individual, his occupation and habits, which determines the seat of the morbid process. The close association of the two diseases is shown by the fact that there are a considerable number of cases in which it is difficult to say whether they should be classed as tabes with mental symptoms, or tabetic general paralysis. About 10 per cent. of cases of general paralysis are associated with tabetic cord lesions. Again the etiological relationship of the two diseases is almost identical; both are affections of men in the prime of life, of townspeople, of men of all grades of society, but women of the lower and lower-middle class only; moreover, juvenile general paralysis and juvenile tabes occur in the subjects of congenital syphilis and affect the sexes almost equally.

There are two types of juvenile general paralysis—(1) those in which the syphilitic poison seemed to have produced an arrest or impaired development of the higher centres, so that the child was weak-minded or imbecile. (2) Those in which the child was mentally capable, in some cases highly intelligent and active-minded. The cause of the degenerative processes in most of these cases seemed to be associated with the increased psychical activity which must of necessity develop after puberty, when new emotions, objects, and ambitions are awakened by the sexual instinct and the struggle for existence. Alcohol materially increases the rapidity of the progress of the disease.

The whole cerebrum and spinal cord waste in general paralysis, but certain regions, viz., the frontal and central convolutions, are earlier and more markedly affected than others. These are the regions whose veins drain into the longitudinal sinus, and in which congestive stasis is liable to occur. If the durability of the nervous system has been lowered, increased psychical activity will tend to exhaustion and disturbance of the normal nutritional equilibrium of the neurones. But psychical activity will cause hyperæmia and congestion of the brain; and in regions where there is a tendency to stasis the congestion may persist, especially if it leads to insomnia. A vicious circle thus becomes established.

The morbid process in general paralysis is probably a primary degeneration of the neurone with secondary meningo-encephalitis, and not the reverse as has been thought, for the following reasons: Its relation to tabes; the existence of the Argyll-Robertson pupil, and of grey atrophy of the optic discs in a number of cases; the wasting of the whole nervous system out of proportion to the evidences of inflammation; in many cases of tabes, especially those with mental symptoms, atrophy of the tangential and super-radial association fibres in the brain are found quite similar to but in different situations from that occurring in general paralysis; and lastly, in diseases with primary meningo-encephalitis we do not find this extreme atrophy.

Of the causes of nervous disease hereditary predisposition stands pre-eminently first. It may be paternal, maternal, convergent, or from grandparents. Thus there are a number of inherited diseases which, though rare, are of great interest, insomuch as they affect members of a family, the disease frequently commencing in each individual at about the same age. Such family diseases are hereditary ataxia (Friedreich's disease), hereditary chorea, amaurotic idiocy, and para myoclonus multiplex. The neuropath may be conceived to possess systems of neurones of defective durability, which, under the influence of contributory factors, such as toxic conditions of the blood or undue stress, undergo premature degeneration. Certain acquired conditions in the parents, affecting them especially at the time of conception, are liable to produce this defect in the germinal plasma. Thus, in the case of juvenile general paralysis, 80 per cent. had an undoubted history or signs of congenital syphilis, and in the other 20 per cent. it could not be excluded. Again, alcoholism is often a most potent cause of nervous degeneration; and in this connection there is a most curious fact—although at least 20 per cent. of people admitted to London county asylums show a history of intemperance, yet cirrhosis of the liver is very rare at autopsies. One must suppose, then, that long before these people could drink sufficient to produce cirrhosis of the liver, symptoms of alcoholic poisoning of the central nervous system arose. A person who could drink sufficiently to produce a hob-nailed liver has inherited a nervous system of unusual stability. To the mentally unstable alcohol is a poison. Epilepsy, insanity, loss of moral control and will power, imbecility, and mental weakness are frequently the heritage of children born of drunken parents and chronic tipplers. The two most important extrinsic causes of degeneration of the neurone are thus syphilis and alcohol.

[2] Broadbent, Lancet, Jan. 27, 1900. [3] Bradbury, System of Med., Vol. vii., 1900. [4] Mott, Brit. Med. Jour., July 7, 1900.

